# Risk of Alzheimer’s Disease and Related Dementia Among Adults With Congenital and Acquired Disabilities

**DOI:** 10.1093/geroni/igab046.875

**Published:** 2021-12-17

**Authors:** Elham Mahmoudi, Paul Lin, Neil Kamdar, Anam Khan, Mark Peterson

**Affiliations:** 1 University of Michigan, Commerce Township, Michigan, United States; 2 University of Michigan, Ann Arbor, Michigan, United States; 3 University of Michigan School of Public Health, Ann Arbor, Michigan, United States; 4 University of Michigan, University of Michigan, Michigan, United States

## Abstract

Objective: Adults with congenital (cerebral palsy or spina bifida (CP/SB)) or acquired disabilities (spinal cord injury (SCI) or multiple sclerosis (MS)) have higher incidence of age-related health conditions. There is a gap in the literature about the risk of dementia among adults living with these disabilities. This study aimed to examine time to incidence of Alzheimer’s disease and related dementia (ADRD) among these disability cohorts. Method: Using national private payer claims data from 2007-2017, we identified adults (45+) with diagnosis of CP/SB (n=7,226), SCI (n=6,083), and MS (n=6,025). Adults without disability diagnosis were included as controls. Using age, sex, race/ethnicity, cardiometabolic, psychologic, and musculoskeletal chronic conditions, and socioeconomic variables, we propensity score matched persons with and without disabilities. Incidence of ADRD was compared at 4-years. Cox Regression was used to estimate adjusted hazard ratios (aHR) for incident early and late onset ADRD. Results: Incidence of early and late onset ADRD were substantially higher among people with disabilities compared to their non-disabled counterparts. Adults with CP, SCI, and MS had higher risk for early [CP/SB: aHR= 3.35 (95% CI: 2.18-5.14); SCI: aHR=1.93 (95% CI:1.06-3.51); and MS: aHR=4.49 (95% CI:2.62-7.69)] and late [CP: aHR=1.68 (95% CI:1.38-2.03); SCI: aHR: 1.77 (95% CI:1.55-2.02); and MS: aHR=1.26 (95% CI:1.04, 1.54)] onset ADRD. Conclusions: Risk of ADRD was higher among adults with CP/SB, SCI, and MS compared to their matched cohort without disability. Investment in early screening and use of therapeutic or rehabilitative services that may help preserving cognitive function among these patient cohorts is warranted.

